# Hypertriglycéridémie majeure sous tamoxifène

**Published:** 2012-11-28

**Authors:** Youssef Khabbal, Hanane El Ouahabi, Salma Bensbaa, Loubna Agerd, Sami Brahmi, Omar El Mesbahi, Farida Ajdi

**Affiliations:** 1 Service ďEndocrinologie Diabétologie et Nutrition, CHU Hassan II, Fès, Maroc; 2 Service de Pharmacologie, Laboratoire Central ďAnalyses Médicales, CHU Hassan II, Fès, Maroc; 3 Service ďOncologie Médicale, CHU Hassan II, Fès, Maroc

**Keywords:** Hypertriglycéridémie, tamoxifène, pharmacovigilance, hypertriglyceridemia, tamoxifen, pharmacovigilance

## Abstract

Plusieurs agents pharmacologiques sont incriminés dans les hyperlipidémies. Le tamoxifène fournit un exemple de médicament hypertriglycéridémiant. Nous décrivons ici le cas ďune patiente atteinte de cancer du sein traitée par tamoxifène qui a présenté une hypertriglycéridémie sévère. Nous présentons aussi une brève revue de la littérature et discutons les mécanismes sous jacents aux effets hypertriglycéridémiants du tamoxifène.

## Introduction

Divers médicaments ont été incriminés dans la survenue d'hypertriglycéridémie: les corticoïdes, le traitement antirétroviral, les antipsychotiques, les diurétiques thiazidiques. Des cas d'hypertriglycéridémie ont été rapportés sous traitement par tamoxifène qui est un anti-oestrogène de synthèse utilisé depuis les années 1970 dans le traitement adjuvant du cancer du sein hormono-dépendant [1], parmi ses effets indésirables, une action sur le métabolisme lipidique [2].

Des cas d'hypertriglycéridémie majeure ont été publiés dans la littérature dont la plupart chez des patients ayant comme facteur de risque une dyslipidémie. L'originalité de cette observation est l'apparition d'une hypertriglycéridémie majeure sous traitement pat tamoxifène conduit comme traitement adjuvant pour cancer du sein chez une patiente sans antécédents de dyslipidémie.

## Patient et observation

Nous rapportons le cas clinique d'une patiente âgée de 44 ans, opérée pour un cancer du sein en 2008, qui a subi une radiothérapie et qui a été traité par antiestrogène le tamoxifène citrate à la dose de 20 mg/j, le délai d'exposition a été une dizaine de mois. La patiente était indemne de toute dyslipidémie avant traitement; dans ses antécédents familiaux la patiente présente une hérédité diabétique sans notion de dyslipidémie.

Le bilan réalisé dans le cadre de la surveillance montrait une hypertriglycéridemie majeure à 10 g/l: intervalle valeur normale (0,6-1,5), le taux de HDL a été bas à 0,2 g/l (0,4-0,7). La glycémie à jeûne était normale: 1,06g/l (0,7-1,1) et le contrôle des enzymes pancréatiques s'avérait sans anomalie.

Les hypertriglycéridémies secondaires ont été évoquées, mais devant la normalité de la glycémie, du bilan thyroïdien, et du bilan rénal ainsi que l'absence d'obésité, de syndrome métabolique ou de prise d'alcool; l’étiologie médicamenteuse a été retenue après étude d'imputabilité selon la méthode de BEGAUD.

Par ailleurs, le dosage de la tamoxifénémie n'a pas pu être réalisé vu qu'il n'est pas disponible au Maroc. La patiente a bénéficié d'une consultation diététique avec suppression des sucres rapides et mise sous fénofibrate 160 mg/j comme traitement symptomatique avec nette amélioration des triglycérides et retour à la normale après 2 mois de traitement, malgré que le tamoxifène n'ait pas pu être arrêté au vue de l’évolutivité de la maladie.

## Discussion

L'hypertriglycéridémie est une élévation des triglycérides au-delà de 1,50g/l (1,7 mmol/l), elle peut être primitive ou secondaire à un diabète, une hypothyroïdie, un syndrome métabolique, voire une prise médicamenteuse. L'hypertriglycidémie majeure se définit par un taux de triglycérides plasmatiques au delà de 10 g/L.

Des cas d'hypertriglycéridémie majeure sous Tamoxifène ont été publiés dans la littérature (Tableau 1), il semble que le Tamoxifène stimule la synthèse et la sécrétion par le foie des lipoprotéines de très faible densité (VLDL), qui représente le principal transporteur de triglycérides (TG) [5], et qui diminue le catabolisme des lipoprotéines de densité intermédiaire (IDL) et des lipoprotéines de très faible densité (VLDL), par diminution de l'activité de la lipoprotéine lipase et triglycéride (TG) lipase hépatiques, qui sont des enzymes clés dans le catabolisme des TG, et qui en résulte une augmentation des TG. Le délai d'apparition des anomalies lipidiques est de quelques mois voire des années après le début du traitement par le tamoxifène.

**Tableau 1:** Cas d'hypertriglycéridémie sous tamoxifène rapportés dans la littérature

Auteur	Age (années)	Antécédents de Dyslipidémie	Triglycéridémie (mg/dl)	Délai d'apparition (mois)
Noguchi et al [3]	34	Non précisé	3673	7
Colls et al [4]	44	Oui	6984	Non précisé
Elisaf et al [5]	53	oui	5200	8
Artac et al [6]	51	Non précisé	1344	12
Lin et al [7]	43	oui	1040	24
Alagozlu et al [8]	46	oui	900	12
Sakhri et al [9]	44	oui	1180	12
Brun et al [10]	61	oui	2790	NP

L'hypertriglycéridémie se voit surtout chez les patients dyslipidémiques initialement ou à antécédents familiaux de dyslipidémie, les patients normolipémiques ont une capacité intacte à cataboliser les lipoprotéines et présentent une élévation modérée des TG.

Le traitement de l'hypertriglycéridémie sous tamoxifène repose comme toute autre dyslipidémie sur les mesures hygiéno-diététiques avec suppression des sucres rapides, et l'utilisation des médicaments hypolipémiants [8], et arrêt du tamoxifène en concertation avec le médecin traitant, avec généralement retour à des valeurs normales de TG. Chez notre patiente nous n'avons pas eu recours à l'arrêt du médicament après discussion avec son oncologue traitant.

## Conclusion

L'hypertriglycéridémie sous tamoxifène reste une complication peu connue, se voyant surtout chez les patients dyslipidémiques ou à antécédents familiaux de dyslipidémie, et dans de rares cas chez les patients normolipidiques. Ceci souligne l'intérêt de la surveillance du bilan lipidique [4, 5, 10] au cours de ce traitement.
